# A Case Series With Cysteamine–Isobionicamide Complex: Clues for Skin‐Rejuvenating Activity

**DOI:** 10.1111/jocd.16743

**Published:** 2025-01-16

**Authors:** Lesley Clark‐Loeser, Riccardo Sfriso, Laure Dirlewanger, Behrooz Kasraee

**Affiliations:** ^1^ Precision Skin Institute Davie Florida USA; ^2^ Scientis SA Geneva Switzerland

**Keywords:** anti‐aging, Cysteamine, hyperpigmentation, Isobionic‐amide, lentigines, photoaging

## Abstract

**Background:**

Skin aging is inevitable. Wrinkles, skin texture abnormalities, senile hyperpigmentation, loss of skin tone, dryness, atrophy, and telangiectasias represent some of the hallmarks of aged skin. Skin rejuvenation can be addressed by topical therapies, such as topical retinoids and antioxidants or physical modalities with energy‐based devices, all providing acceptable outcomes. In this case series, we aimed to test the rejuvenating potential of the combination of cysteamine (a naturally occurring antioxidant) and isobionicamide (a derivative of the anti‐aging molecule niacinamide) applied topically.

**Methods:**

Healthy male and female patients (*N* = 7) aged between 25 and 70 years and having Fitzpatrick skin types I–VI were recruited. Topical application of a cysteamine‐isobionicamide formula was done once daily. Treatment lasted for 16 weeks. Clinical high‐resolution photos were acquired using LifeViz 3D at recruitment and after 16 weeks. Blinded dermatological examinations and scoring were performed. Self‐assessment and quality of life (QoL) questionnaires were collected.

**Results:**

Clinical photos showed improvement in skin luminosity, increased evenness of skin tone, and reduction of fine wrinkles as well as hyperpigmentation. Patients as well as clinical investigators blinded to the chronology of photos observed the improvements in skin texture, luminosity, and radiance, the brightening of the dark spots, as well as the reduction of both number and volume of wrinkles after 16 weeks of daily application. Furthermore, a significant improvement in patients' quality of life was recorded.

**Conclusion:**

This case series represents the first evidence that topical application of cysteamine isobionic‐amide complex could be considered as a safe and effective option in the reversal of skin photoaging.

## 1 Introduction

Skin aging is the result of the interaction of genetic and non‐genetic (defined as exposomal) factors. The latter including chronic, life‐long exposure to sunlight affecting both keratinocytes and fibroblasts, leading to several molecular changes resulting in the breakdown of collagen in the extracellular matrix and downregulation of new collagen synthesis [1]. These molecular changes underlie the development of premature skin aging (photoaging). Clinically, chronic photoaging may result in functional and structural alterations to the skin, such as the appearance of fine wrinkles, skin texture abnormalities, mottled pigmentation (hypo‐ or hyperpigmentation), loss of skin tone, dryness, severe atrophy, telangiectasias, and actinic keratoses. Furthermore, ultraviolet radiation (UVR) exposure impacts skin's antioxidants. Ascorbate, glutathione (GSH), superoxide dismutase (SOD), catalase, and ubiquinol are depleted in all layers when skin is exposed to UVB [2]. Microscopically, photoaged skin displays a reduction in epidermal thickness, heterogeneous pigmentation, enhanced collagen degradation by increased expression and activity of metalloproteinases, as well as increased generation of reactive oxygen species (ROS), leading to mutagenesis in melanocytes and keratinocytes [1].

In recent years, many new products, therapies, and procedures have arisen aiming at minimizing the erythema, dyspigmentation, and rhytides associated with photoaged skin. Skin resurfacing through the complete ablation of layers of skin was one of the initial procedures developed for facial rejuvenation. Because of its efficacy, ablative laser resurfacing is still considered the gold standard for facial skin rejuvenation. However, significant patient downtime and the risk of adverse events, such as scarring and skin dyspigmentation, are some of the downsides of this technique. Fractional resurfacing represents an intermediate approach that only ablates micro‐columns of epidermal and dermal tissue over a fraction of the skin surface, providing a faster recovery as compared to ablative laser resurfacing. Last, non‐ablative procedures targeting dermal collagen without damaging the epidermis became available. Among these, intense pulsed light is the most commonly used, as it effectively targets both erythema and dyspigmentation seen in photoaging [3]. Although non‐ablative techniques are able to successfully minimize the side effects and patient downtime, they are far from matching the results of ablative procedures.

Topicals aim to either prevent ultraviolet damage via photoprotection (e.g., sunscreen) or help reduce and reverse existing signs of photoaging in order to restore the skin to its highest functioning level. Research of effective and safe topical therapies is constantly ongoing, even though topical retinoids, such as tretinoin, isotretinoin, and tazarotene, have been shown in several randomized clinical trials to provide benefits for the treatment of mild to moderate photodamaged skin [4]. Kligman and colleagues in 1984 were the first to demonstrate the efficacy of tretinoin in the treatment of photoaging in an animal model [5]. Later on, Fisher et al., in 1996, observed that pretreatment of photoaged skin with a cream containing 0.1% tretinoin resulted in a complete inhibition of the synthesis of both collagenase and gelatinases, thus preventing collagen degradation [6]. Despite being very efficacious, topical retinoid therapy is not devoid of potential side effects. The most common and frequent adverse reaction to retinoids is the so‐called “retinoid reaction,” characterized by pruritus, burning sensation, erythema, and peeling. Another side effect is photosensitization. Thus, patients under retinoid therapy are advised to avoid excessive sun exposure and use high‐SPF sunscreen.

Fighting the UVR‐induced oxidative stress by topical application of antioxidants, such as vitamin C or vitamin E, is another approach that has also been shown to have beneficial effects on the reversal of photoaged skin. Ascorbic acid (Vitamin C), well‐known for its antioxidant properties, has been shown to upregulate collagen synthesis and prevent UVR‐induced erythema [7]. Several studies reported that topical application of products containing different concentrations of Vitamin C (ranging from 5% to 17%) resulted in clinically significant skin restoration from photoaging signs with both improvements in skin texture, skin tone, and reduction of wrinkles [7, 8]. Nevertheless, the main drawbacks of ascorbic acid are its stability and poor cutaneous penetration.

Pigmentary alterations are among the major features of photoaged skin and may be observed in all skin types. Cysteamine is a potent endogenous antioxidant found in human tissues and in particularly high concentration in human milk [9]. In 1966, a group of researchers led by Dr. Chavin discovered the depigmenting effects of cysteamine after injecting cysteamine hydrochloride into the skin of a black goldfish and surprisingly found that the skin of the fish turned white [10]. Later on, studies by Dr. Pathak and Dr. Bleehen demonstrated that cysteamine was significantly more effective than hydroquinone in depigmenting the skin of black Guinea pigs [11]. More recently, stabilized cysteamine has become available for topical use. Several studies followed and confirmed its high efficacy for the treatment of hyperpigmentation disorders [9, 12].

**FIGURE 1:**
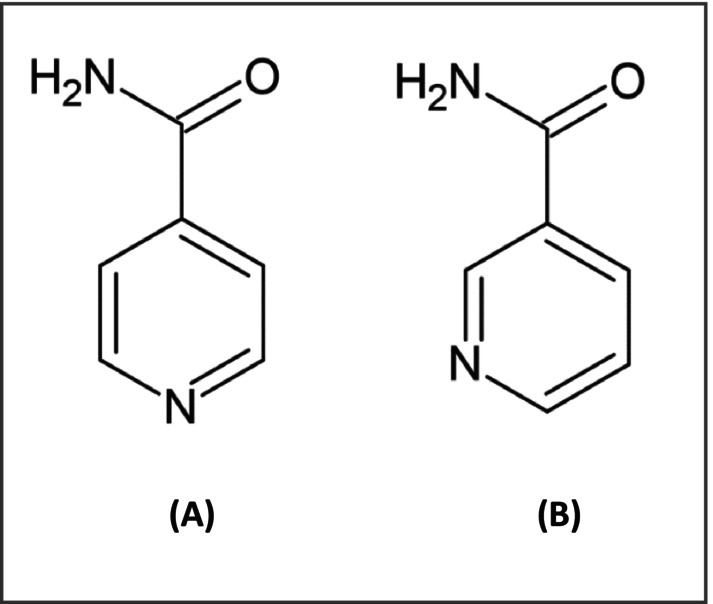
Chemical structure of isobionic‐amide (A) compared to niacinamide (B).

A recent cysteamine‐based formulation including isobionic amide—an isomer of niacinamide shown to be a potent melanosomal transfer inhibitor, with synergistic effect on tyrosinase inhibition when combined with cysteamine [13] (see Figure 1)—has become available to the armamentarium of dermatologists. A recent clinical trial has compared the efficacy of the cysteamine isobionic‐amide complex to that of the gold standard triple combination cream (modified Kligman's formula) for the treatment of melasma. The study showed that the product showed comparable onset of action as early as 4 weeks and equivalent efficacy to the gold standard after 16 weeks with a high tolerability and absence of side effects [13].

Several studies point to the crucial role of reactive oxygen species (ROS) in aging. Hydrogen peroxide (H_2_O_2_) is one of the most important ROS in this regard, and it is shown that H_2_O_2_ not only induces aging at the cellular level, but its increase in the organism causes aging and reduced lifespan in vivo [14]. Cysteamine is a potent, cell‐permeable antioxidant that directly destructs H_2_O_2_ [15] and thus could be theoretically effective as an anti‐aging molecule. In combination with isobionic‐amide, cysteamine showed a potent depigmenting action against skin hyperpigmented lesions [13]. Since hyperpigmentary disorders (e.g., senile lentigo) are part of the skin aging process, the Cysteamine Isobionic‐amide combination was chosen to evaluate its skin anti‐aging properties.

## 2 Materials and Methods

The case series was conducted at Precision Skin Institute, Florida, USA. The protocol of this case series was conducted in compliance with the WHO ICH Harmonized Tripartite Guidelines for Good Clinical Practice and the Declaration of Helsinki. Informed consent was obtained from the patients prior to enrollment. Healthy male and female patients (*N* = 7) aged between 25 and 70 years and having Fitzpatrick skin types I–VI were recruited. The treatment lasted for 16 weeks. The inclusion criteria were: (i) Male and female subjects in good health, and between the ages of 25–70 years old, inclusive, at the time of enrollment; (ii) Fitzpatrick Skin Type I–VI; (iii) Open to all ethnicities; (iv) all skin types (normal, oily, dry, combination); (v) subjects free of any systemic or dermatological disorder, including a known history of allergies or other medical conditions that could interfere with the conduct of the study, interpretation of results, or increase the risk of adverse reactions; (vi) subjects who are free of any skin condition(s) or who have a history of skin condition(s) at the test site that could interfere with the purpose, conduct, or integrity of the study; (vii) subjects that agree not to introduce new products, procedures, or collagen induction therapies for the duration of the study; (viii) subjects will be able to read, understand, and sign an informed consent form (includes HIPAA and State requirements, as appropriate for the respective country); (ix) subjects willing to be photographed and sign a photograph release form; (x) subjects are willing to use the assigned test material and supporting materials as instructed for the duration of the study and do not start using any new facial products other than the assigned test materials; (xi) subjects willing and able to follow all study directions, attend study visits as scheduled, and must be willing to accept the restrictions of the study; (xii) subjects who agree to avoid excessive sun exposure and the use of artificial tanning methods for the duration of the study.

### 2.1 Screening Phase

Patients meeting the inclusion criteria were selected. The dermatologist analyzed the medical history and examined the skin of each patient for any sign of inflammation or irritation. Furthermore, a list of current skin care products was collected for each study participant. The washout phase lasted 14 days, during which patients were asked to discontinue any topical or oral depigmenting treatments and medications. The baseline visit was scheduled after 2 weeks.

### 2.2 Case Series Study Design

At the baseline visit, patients were provided with products and instructions on how to apply them once daily in the morning or in the evening for all the duration of the study. The products provided were a system consisting of: a short‐contact mask (Cyspera Intensive), a cleanser (Cyspera Neutralize), and a leave‐on cream (Cyspera Boost). Patients were instructed to (1) apply on the entire face a thin layer of short‐contact mask (Cyspera Intensive) on unwashed and rested skin once per day in the morning or in the evening; (2) after 15 min apply directly the cleansing product (Cyspera Neutralize) and rinse it off with water; and (3) apply a thin layer of the leave‐on product (Cyspera Boost). All patients were instructed to apply a broad‐spectrum mineral sunscreen, twice daily, in the morning and at mid‐day. Cyspera Intensive, Neutralize, and Boost were provided by Scientis SA (Geneva, Switzerland). Digital high‐resolution images were acquired using LifeViz 3D (Quantificare SA, France). On these, blinded dermatological examination and scoring of overall skin appearance, skin texture, radiance, wrinkles, pigmentation, evenness of skin tone, presence of dark spots, age spots, and rating of the overall improvement were given. On the other side, patients were asked to fill in both a self‐assessment and quality of life (QoL) questionnaires at all timepoints. Monthly phone assessments were done by the dermatologist. Lastly, a cohort of seven volunteers external to the study was asked to identify the pre‐ and post‐treatment pictures by selecting the image in which the subjects appeared younger and by estimating the age difference between the two pictures.

## 3 Results

A very good skin tolerance was determined as the dermatologist did not observe any clinical signs of irritation, dryness, or erythema. The products were considered safe, and no signs of photosensitivity were observed throughout the study.

### 3.1 Skin Health Improvements

**FIGURE 2:**
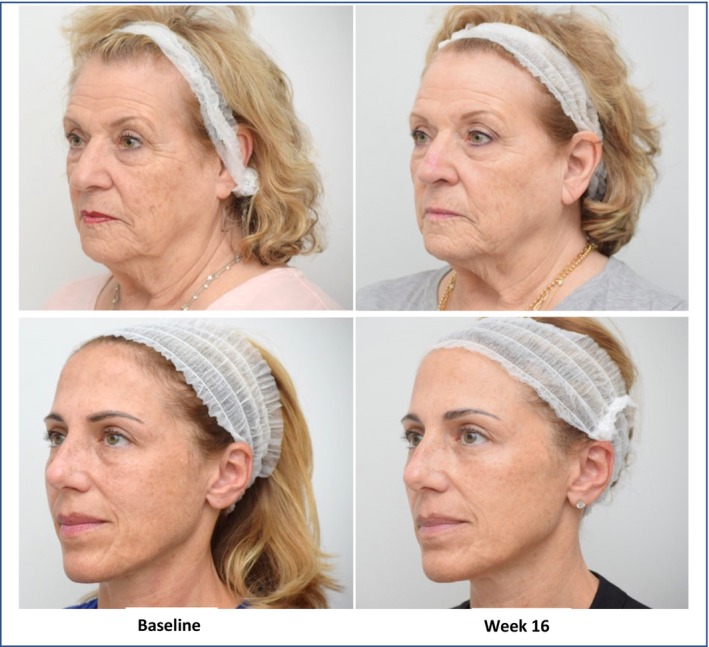
Clinical photos showing the progress of two patients (volunteer 3 on top, volunteer 6 on the bottom) at week 4 and at the end of the study (week 16).

The products showed a fast onset of action with significant and visible results within 2–4 weeks. (Figure 2).

**FIGURE 3:**
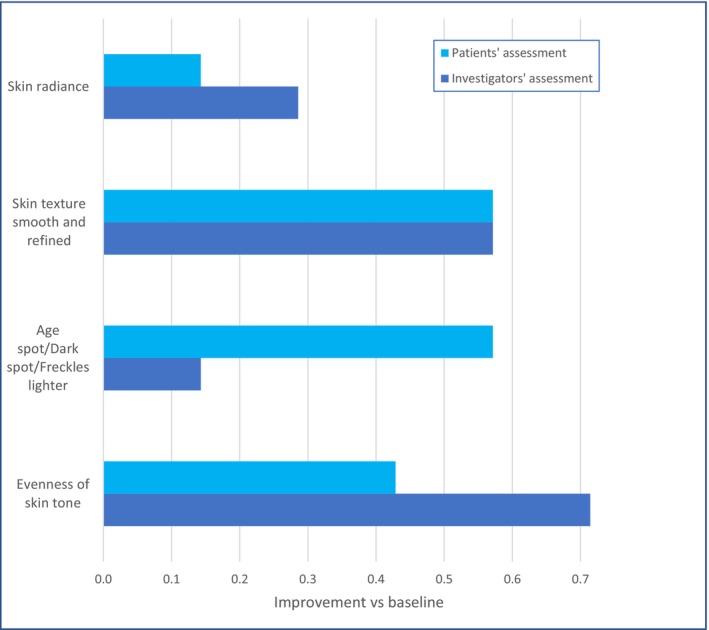
Investigators' assessments and patients' self‐assessments. Independent and blinded dermatologists (*N* = 2) were asked to assess the skin of the patients on the different parameters at two timepoints: Baseline and week 16. Patients (*N* = 7) were asked to fill in the self‐evaluation questionnaire at baseline and at week 16. Showed are average values.

Significant evening of skin tone in terms of uniformity and reduced contrasts was observed. No halo effect was observed in any of the patients. An overall improvement of skin health parameters such as skin radiance, skin tone evenness, skin texture, and brightening of age spots, as well as improved hydration and an overall skin rejuvenation, were observed by the blinded investigators as well as reported by the patients (Figure 3).

**FIGURE 4:**
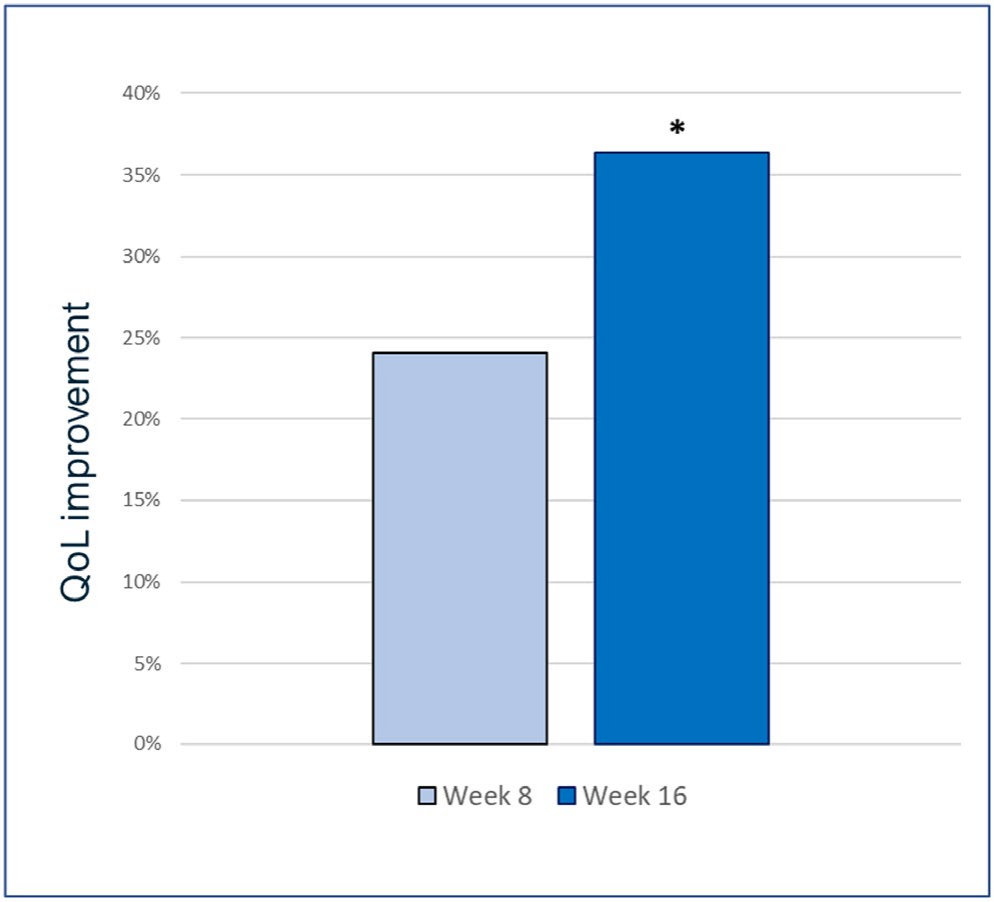
Improvement of Quality of Life (QoL) over 16 weeks of treatment showed as improvement (%) from baseline. *p < 0.05.

The results of the quality of life (QoL) questionnaire highlighted that the quality of life of the patients significantly improved after 16 weeks compared to baseline (*p* < 0.05). (Figure 4).

Presenting randomized pictures of the study subjects before and 16 weeks after the application of the products to 7 volunteers external to the study resulted in 76% of the evaluators identifying the subject of the “after” picture as appearing “younger” than the “before” pictures. Notably, for the volunteers 3 and 6 (see Figure 2), 100% of the evaluators perceived the “after” picture as younger than the “before” picture, with a respective reduction in age of 6.7 and 4.1 years. On average, the subjects in the “after” picture appeared 2.2 years younger than in the photo taken before the start of the study.

## 4 Discussion

The results of this case series suggest that the cysteamine isobionic‐amide complex is effective in improving signs of photoaging when applied topically once daily over the course of 16 weeks.

Based on the ROS‐induced aging theory, several antioxidants have been tested for their ability to scavenge free radicals, thus protecting skin from oxidative stress or even reversing aging signs (Table 1).

**TABLE 1:** Most common exogenous antioxidants.

Antioxidants	Photoprotective effect	Suppression of H_2_O_2_	Cutaneous permeability/Penetration
Ascorbic acid	Topical vitamin C 5% cream applied for six months led to clinical improvement in the appearance of photoaged skin [2]	Not known	Poor
Vitamin E	Complement the photoprotective effects of other antioxidants [2]	Not known	Good
Glutathione	Not shown in vivo [2]	Yes [16]	Poor
Cysteamine	Improvement in the appearance of photoaged skin after 16 weeks of use is shown in this study	**Yes** [15]	**Good**

Among ROS, the role of H_2_O_2_ in the whole process of aging is coming more and more into the spotlight during the last decades. Although the destruction of superoxide ions did not affect the aging process in vivo, H_2_O_2_ reduction through the use of the scavenger protein catalase not only improved the healthspan of aging mice but also increased their lifespan [17]. Very recently, the counteraction with H_2_O_2_‐induced fibroblast damage has been proposed as a novel target for the management of skin aging. However, many antioxidants with H_2_O_2_‐reducing activities in vitro do not possess the necessary properties required to exert their action in an in vivo environment. One of such properties is the ability of the molecule to penetrate into the skin by passing the lipophilic stratum corneum layer (i.e., the barrier against most hydrophilic antioxidants such as ascorbic acid and ferulic acid) and entering into the cytoplasm (i.e., the barrier against lipophilic antioxidants such as tocopherol and retinol). On the other hand, many of the most potent antioxidants in vitro, such as polyphenols, have high molecular weights, prohibiting them from effective skin penetration.

Cysteamine is a potent endogenous antioxidant [9, 15]. Cysteamine is shown, in concentrations higher than 10 mM, to directly remove H_2_O_2_ in cell‐based systems and to protect cells from the H_2_O_2_ load [15]. It is also shown that cysteamine readily penetrates human skin ex vivo [15]. The cysteamine isobionic‐amide combination, shown to be a very effective depigmenting complex, might thus serve as a suitable anti‐aging complex that, on the one hand, removes the skin H_2_O_2_ to counteract the process of aging and, on the other hand, reduces the hyperpigmented lesions that are considered a sign of skin aging.

In our case series, patients as well as blinded investigators noticed improvements in skin texture, luminosity, and radiance; brightening of the dark spots; as well as reduction of both number and volume of wrinkles after 16 weeks of daily application. Furthermore, a significant improvement in patients' quality of life was recorded, likely because of an overall healthier‐looking and younger skin. The clinical photos of the patients allowed a visual comparison with the baseline situation, evidencing improvements in all the above‐mentioned features.

Solar lentigines are one of the hallmarks of photoaged skin. These macular hyperpigmented skin lesions are seen most commonly after the age of 50 and appear on sun‐exposed areas such as the face, neck, hands, and forearms [18]. The first‐line therapy for solar lentigines is ablative therapy with cryotherapy. Physical therapies have the advantage of providing rapid visible results compared with topical therapies, but the former are characterized by significant patient downtime and potential side effects. Topical agents such as glycolic acid [19], tretinoin [20], and hydroquinone [21, 22] are typically not as effective as the physical therapies in depigmenting solar lentigines. Topical cysteamine has been shown to significantly reduce the hyperpigmentation of solar lentigines even in the absence of retinol and glycolic acid [13]. The clinical photos in this case series showed a visible skin lightening and evenness after 16 weeks of product use, corroborating the effective depigmenting action of the cysteamine isobionic‐amide formula.

It is reported that iron accumulates in the body, and especially in the skin, during chronological aging [23, 24]. Skin is constantly exposed to oxygen and to the oxidizing component of sunlight; both are detrimental to iron homeostasis and contribute to accelerating aging processes. Exposure to UV light causes degradation of ferritin, the major iron‐storage protein, which, as a result, releases free iron inside skin cells. This “free” iron may act as a catalyst in reactions between reactive oxygen species and biomolecules, leading to increased oxidative stress, exacerbation of skin pigmentation, and ultimately skin photoaging [24, 25]. Cysteamine is known to chelate metal ions, such as iron and copper, as well as hydroxyl ions resulting from Fenton‐type reactions, and this is one of the mechanisms through which it indirectly inhibits the melanogenesis pathway [10]. We speculate that iron chelation and reduction of oxidative stress could be additional mechanisms through which the tested cysteamine‐based product delivered the antiaging effects observed in the current case series, although additional studies will be needed to further corroborate this hypothesis. In addition, it is likely that other molecules present in the tested formulation, such as retinol and vitamin C derivatives, such as ascorbyl palmitate and sodium ascorbyl phosphate, could have contributed, in concert with cysteamine, to the observed rejuvenating effects.

The limitations of this case series obviously reside in the limited number of patients, a wide age range, and the inclusion of both genders. Additionally, the case series is constrained by a limited amount of quantitative data due to the absence of objective measurements for comparison. Larger studies are needed to understand more in‐depth mechanisms of action underlying the observed rejuvenating effects; however, we believe that our case series helps shed some light on the rejuvenating potential of the topical cysteamine isobionic‐amide complex.

## 5 Conclusion

This case series represents the first evidence that topical application of the cysteamine‐isobionicamide complex could be considered as a safe and effective candidate in the reversal of skin photoaging. The product may be used as well in conjunction with in‐office procedures such as ablative and non‐ablative laser therapies, acid peel resurfacing, or microdermabrasion. However, larger studies with a more homogenous study group in terms of age and gender, and including comparative analysis with quantitative and objective measures, would be needed to confirm the photoaging effects of the cysteamine‐isobionicamide complex.

## Author Contributions

All authors have read and approved the final manuscript. L.C.‐L. performed the study conception and study design, the patient recruitment, the patient diagnosis, and the collection. and analysis of the data. R.S., L.D., and B.K. wrote the manuscript.

## Ethics Statement

Written informed consent was obtained from the patient for publication of this case report and accompanying images.

## Conflicts of Interest

L.D., R.S., and B.K. are employees of Scientis SA, but did not participated in the patient recruitment, patient diagnosis, the data collection, and their analysis. The remaining authors have no conflicts of interest to declare.

## Data Availability

The data that support the findings of this study are available from the corresponding author upon reasonable request.
